# Advancing the Spatiotemporal Dimension of Wildlife–Pollution Interactions

**DOI:** 10.1021/acs.estlett.5c00042

**Published:** 2025-03-18

**Authors:** Jack A. Brand, Jake M. Martin, Marcus Michelangeli, Eli S.J. Thoré, Natalia Sandoval-Herrera, Erin S. McCallum, Drew Szabo, Damien L. Callahan, Timothy D. Clark, Michael G. Bertram, Tomas Brodin

**Affiliations:** † Department of Wildlife, Fish, and Environmental Studies, Swedish University of Agricultural Sciences, Umeå 907 36, Sweden; ‡ Institute of Zoology, Zoological Society of London, London NW1 4RY, United Kingdom; § Department of Zoology, Stockholm University, Stockholm 114 18, Sweden; ∥ School of Biological Sciences, Monash University, Melbourne 3800, Australia; ⊥ School of Life and Environmental Sciences, Deakin University, Waurn Ponds 3216, Australia; # Australian Rivers Institute, Griffith University, Nathan 4111, Australia; ∇ TRANSfarm - Science, Engineering, & Technology Group, KU Leuven, Lovenjoel 3360, Belgium; ○ Laboratory of Adaptive Biodynamics, Research Unit of Environmental and Evolutionary Biology, Institute of Life, Earth and Environment, University of Namur, Namur 5000, Belgium; ● Centre of Excellence in Mass Spectrometry, Department of Chemistry, University of York, York YO10 5DD, United Kingdom; □ School of Chemistry, The University of Melbourne, Melbourne 3010, Australia

**Keywords:** behavioral ecotoxicology, ethology, field toxicology, landscape ecotoxicology, movement ecology

## Abstract

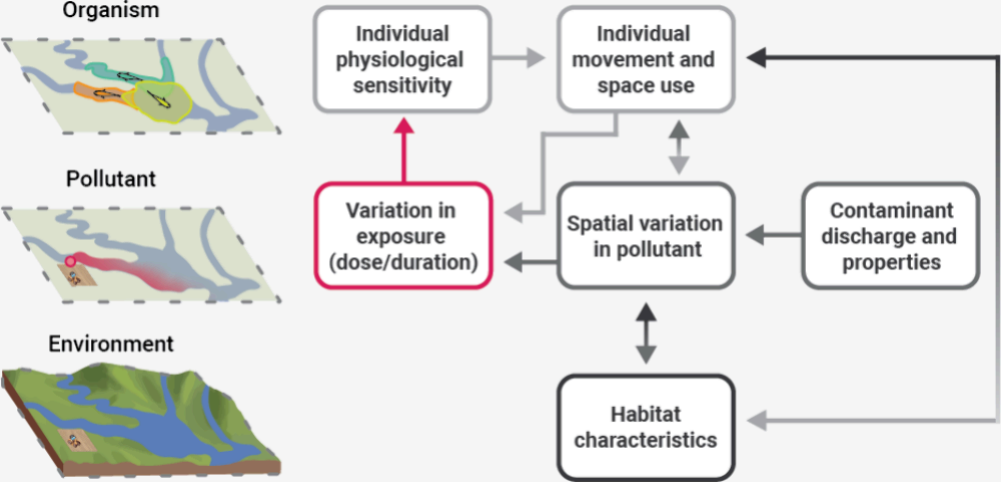

Chemical pollution is one of the fastest-growing agents of global change. Numerous pollutants are known to disrupt animal behavior, alter ecological interactions, and shift evolutionary trajectories. Crucially, both chemical pollutants and individual organisms are nonrandomly distributed throughout the environment. Despite this fact, the current evidence for chemical-induced impacts on wildlife largely stems from tests that restrict organism movement and force homogeneous exposures. While such approaches have provided pivotal ecotoxicological insights, they overlook the dynamic spatiotemporal interactions that shape wildlife–pollution relationships in nature. Indeed, the seemingly simple notion that pollutants and animals move nonrandomly in the environment creates a complex of dynamic interactions, many of which have never been theoretically modeled or experimentally tested. Here, we conceptualize dynamic interactions between spatiotemporal variation in pollutants and organisms and highlight their ecological and evolutionary implications. We propose a three-pronged approach—integrating *in silico* modeling, laboratory experiments that allow movement, and field-based tracking of free-ranging animals—to bridge the gap between controlled ecotoxicological studies and real-world wildlife exposures. Advances in telemetry, remote sensing, and computational models provide the necessary tools to quantify these interactions, paving the way for a new era of ecotoxicology that accounts for spatiotemporal complexity.

## 1 Introduction

Chemical pollution is a globally pervasive problem. The contamination of ecosystems with synthetic chemicals is now considered the fastest-growing agent of global environmental change, with fears that humanity has exceeded the safe operating limits of the planetary boundary for novel entities in the environment.^1−3^ To date, over 350000 chemicals (e.g., plastics, pesticides, pharmaceuticals) are registered for use worldwide,^4^ with an increasing number of these substances being routinely detected in the environment.^5^ Importantly, many of these contaminants have been shown to disrupt wildlife behavior, alter ecological interactions, and shift evolutionary trajectories.^5,6^ Given their widespread presence and capacity to disturb key ecological processes, understanding how pollutants affect wildlife populations remains a critical topic of research.

Over the last several decades, increased environmental monitoring of contaminants has demonstrated that chemicals are often spatially and temporally structured within the environment.^7,8^ In light of this, it has long been acknowledged that spatiotemporal information must be better integrated into ecotoxicology in order to accurately predict a species’ local exposure risk (e.g., landscape ecotoxicology^9−11^). However, our current knowledge of chemical pollution-induced effects on wildlife is largely based on tests performed under simplified laboratory conditions, where the potential impacts of a contaminant are often assessed using a single isolated individual, at one or more set dosages.^12,13^ In most cases, these studies aim to achieve homogeneous exposure conditions—both spatially and temporally—and restrict the physical space in which the study organisms can move. Consequently, there is an underlying assumption that the effects seen under these conditions would be reflective of exposures in the wild.^10^

Like pollutants, organisms are distributed nonrandomly throughout their environment, and their distribution can change over time. Recent high-resolution tracking studies on wild organisms have demonstrated that seemingly similar species, populations, and even individuals within those populations often consistently differ in their movement, space use, and habitat selection,^14−17^ suggesting that organisms differ from one another in their likelihood of encountering pollutants. Furthermore, exposure to chemical pollutants has itself been shown to alter organismal behavior and movement rates,^6,12,18,19^ generating the potential for dynamic feedback loops between spatiotemporally structured chemical pollutants and variation in animal movement.^20^ Given that spatiotemporal dynamics are fundamental to all ecological and evolutionary processes, understanding how the spatial and temporal structuring of contaminants and organisms affects variation in exposure rates, subsequent organismal movement, and how this may scale up to population-level processes is a vitally important area for future research.

We contend that accurately measuring and forecasting the risk of environmental contaminants on wildlife populations depends on (I) the spatiotemporal variation of pollutants, (II) the spatiotemporal variation of organisms, and (III) the relationship between the two. Here, we briefly examine how spatiotemporal variation in pollutants and individual organisms may result in differential exposure risk within populations. We then propose a series of dynamic interactions that could arise from these spatiotemporal processes and discuss how they may scale up to have substantial ecological and evolutionary effects. Finally, we outline promising directions for future research, emphasizing recent advances in analytical chemistry, animal-tracking technologies, and computer-based modeling as a much-needed window into the spatiotemporal elements of environmental ecotoxicology.

## 2 Pollutants Are Spatially and Temporally Structured within Environments

Chemical pollutants are not evenly distributed in the environment across space or time. First, the source of contamination plays a significant role in the spatial distribution of a pollutant. Some pollutants originate from localized point sources, such as wastewater or stormwater outflows, while others result from diffuse sources, such as large-scale agriculture spray-drift. In aquatic systems, factors like water flow patterns, river discharge, and precipitation levels can dilute/concentrate and transport these contaminants once they enter the environment (e.g., ref (21)). For example, the concentration of point-source contaminants typically decreases with distance from the discharge site, as seen with higher zinc contamination in waterways near urbanized areas in Vietnam.^22^ Similar patterns are evident in terrestrial systems, with prior studies showing that contamination of dust and air with pesticides is highest near agricultural lands and is diluted further from the source.^23^ However, it is important to note that this is not always the case, particularly when complex contaminant drift dynamics are involved.^23,24^ Indeed, nonpoint source contaminants, such as agricultural runoff and atmospheric deposition, often show more varied spatial distributions.^25^

The matrix through which contaminants move (e.g., soil, water, or gas), as well as the physical, structural, and molecular properties of chemicals—such as hydrophobicity, functional groups, reactivity, and volatility—also determine their mobility, transformation, persistence, and subsequent distribution in the environment.^25^ Additionally, habitat and environmental characteristics like UV exposure, temperature, precipitation, soil-sediment composition, prevailing wind direction, and ocean currents can influence the degradation and dispersal of contaminants.^8,21,26^ For example, research has shown that sediment type is associated with pollutant hotspots in lakes,^21^ while soil pesticide concentrations can be influenced by physical soil characteristics^26^ and local agricultural practices^27^ in terrestrial systems. Plants, microbes, and animals can further alter contaminant breakdown and distribution through uptake, biomagnification, and biotransformation. These processes can occur across the aquatic–terrestrial interface, where pollutants may be transferred and even biomagnify through trophic interactions between ecosystems.^28,29^

Temporal changes in the spatial distribution of chemical contaminants are also common. For example, a known hotspot of wastewater-derived pharmaceuticals and other pollutants in Lake Geneva, Switzerland, dissipated with a change in thermal stratification in colder months, resulting in a more homogeneous vertical distribution in the water column.^8^ Similarly, seasonal variation in the concentration of pesticides and polycyclic aromatic hydrocarbons (PAHs) has been documented in the Henares River basin in central Spain, likely due to seasonality in agricultural practices and changes in sunlight intensity affecting chemical degradation.^30^ Temporal changes can also occur on much shorter time scales. For example, concentrations of illicit drugs and their metabolites can increase in wastewater following public events.^31,32^ In Lake Qingshan, China, organic pollutant concentrations spiked immediately following heavy rainfall events before eventually declining,^33^ whereas daily variation in the concentrations of organic and heavy metal pollutants in surface waters of the Mekong Delta, Vietnam, were linked to water mixing caused by tidal activity.^22^

The spatiotemporal variation in exposure to chemical pollutants has gained increasing attention.^9,10^ For example, in the Athabasca Oil Sands Region of Canada, recent research integrating spatial geographic information systems with mercury bioaccumulation data—including from amphibians, bird eggs, plants, and terrestrial and aquatic mammals—has identified spatial “hotspots” of mercury contamination near industrial facilities.^34^ Further, in the Puget Sound Basin (Pacific Northwest of the United States), coho salmon (*Oncorhynchus kisutch*) mortality has been linked to nearby road density and traffic intensity, a finding attributed to tire wear particle leachates in urban runoff.^35,36^ However, much of this research has focused on relatively large spatial scales to identify how contaminant exposure varies between species or populations in different locations across time, with little attention paid to how the spatial structuring of these chemicals affects within-population differences in exposure rates, how exposure can subsequently feed back to alter animal movement and space use, and how this may influence broader ecological and evolutionary processes.

## 3 Individuals Are Spatially and Temporally Distributed within Environments

It is well-known that the distribution of organisms varies across both space and time. The movement of animals within their environment, for example, allows species to track changes in resources (e.g., food, breeding sites) and avoid unfavorable environmental conditions. This can occur at large spatial scales over long timeframes (e.g., seasonal shifts in distribution during long-distance migrations), as well as much smaller scales where organisms vary their within-environment space use over shorter timeframes. For example, Eurasian perch (*Perca fluviatilis*) displayed the highest activity rates and increased space use during the day,^37^ while large marine predators like Atlantic bluefin tuna (*Thunnus thynnus*) are also known to migrate hundreds of meters of vertical distance each day, traversing stratified layers of water with remarkably different abiotic profiles.^38^

Individuals within populations also often differ in their space use and movement dynamics.^39^ For instance, individual phenotypes (e.g., body size, body condition, sex, age) have been found to influence movement and space use in a variety of species.^40−45^ Even when controlling for these factors, individuals within populations still often inherently differ from one another in their movement.^15,46^ Indeed, a long-term (8-year) radio telemetry study tracking over 500 individual fish from 5 different species showed that inherent individual differences within populations accounted for more variation in movement dynamics than differences between the tested species.^47^ This intraspecific variation can have key effects on organismal ecology, with previous research showing relationships between individual movement rates, dietary niche, and habitat selection.^14,16,43^ Collectively, this research demonstrates that individuals within populations exhibit significant variation in space use and movement, which are closely linked to niche specialization. Consequently, individual differences may lead to unique patterns of exposure to environmental challenges such as pollutants.^48−51^

## 4 Wildlife–Pollution Interactions in a Spatiotemporal Context

**Figure 1:**
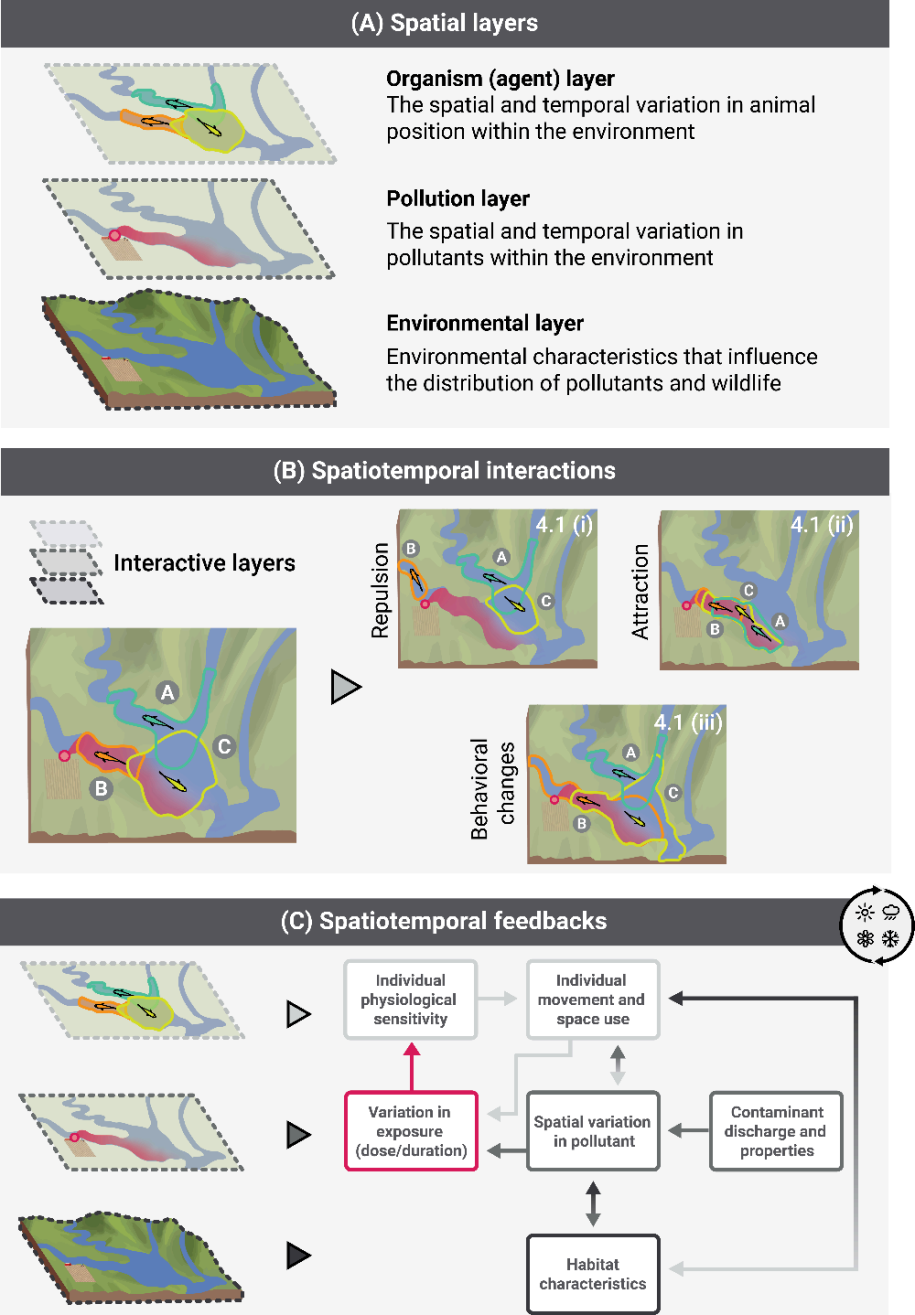
(A) Spatial layers that will influence the exposure risk and outcomes for wildlife. (B) Broad spatiotemporal wildlife–pollutant interactions and possible effects on the individual movement of fish from a hypothetical population. (C) Simple framework outlining potential pathways and dynamic feedback mechanisms between spatiotemporal variation in pollutants and animals that are described in this paper (the shading of the arrows helps represent the spatial layers that are influencing one another). The “seasonal” symbol in the top right-hand side of (C) represents the importance of changing environmental variables in determining spatiotemporal wildlife–pollutant interactions (e.g., via effects on habitat characteristics [ice versus free-flowing river], contaminant discharge rates [seasonal changes in agricultural practices or rainfall patterns], individual space use [seasonal differences in foraging areas or dispersal]).

Given that both pollutants and animals vary in their spatial and temporal distribution within the environment, an individual’s movement patterns, habitat preference, and space use will directly influence its exposure to chemical pollutants. This has been demonstrated in species such as chinook salmon (*Oncorhynchus tshawytscha*),^50^ Pacific bluefin tuna (*Thunnus orientalis*),^51^ Pacific herring (*Clupea pallasi*),^49^ and striped bass (*Morone saxatilis*).^48^ In the wild, an individual’s “realized exposure” is determined by the alignment between its spatiotemporal distribution and that of a pollutant, combined with individual bioaccumulation processes (i.e., the balance of uptake and loss). Importantly, pollutant exposure can also create feedback effects that influence future movement and decision-making, either by disrupting normal behaviors or by triggering avoidance, attraction, or conformity to polluted habitats.^20,52−57^ Below, we conceptualize the dynamic feedback between the spatiotemporal distribution of contaminants and wildlife and discuss the likely ecological and evolutionary consequences (Figure 1).

### 4.1 Pollutants Impact the Spatiotemporal Distribution of Organisms and the Nature of Their Exposure

#### 4.1.1 Wildlife–Pollutant Repulsion-like Interactions

Organisms may actively avoid contaminated areas, with contaminants directly triggering sensorial repellence or by making environments less suitable for occupation (i.e., acting as habitat disrupters).^58,59^ Such effects can alter the duration and extent of individual exposure. For example, even at low concentrations, copper pollution has been shown to induce spatial repellence in numerous taxa (including invertebrates, fish, and amphibians)^60−63^ and can act as a chemical barrier preventing recolonization of suitable habitats and potentially isolating populations.^64^ Organisms may also employ temporal avoidance strategies, especially when displacement is impossible, such as delaying colonization—exemplified by deterred oviposition in polluted habitats^65,66^—or entering dormant stages.^67^ These avoidance behaviors have been demonstrated in laboratory-based, multicompartmental exposure systems^68,69^ and are influenced by the organism’s ability to detect the pollutant (sensory physiology), its capacity to escape (e.g., sessile versus mobile stage, pollutant-induced locomotion impairment), and also environmental features such as resource availability, interspecific interactions, and the characteristics of the chemical exposure (e.g., chemical properties, concentration, and duration).^70^ An important aspect to consider when evaluating risk is that the repellent nature of a substance may not be directly correlated with its toxicity, meaning that a highly repellent contaminant could have low toxicity and *vice versa*.^71^ Moreover, because pollutant-induced spatial avoidance occurs at sublethal concentrations or concentrations too low to produce detectable physiological effects, environmental risk assessments based solely on these measures may overlook important shifts in population and community dynamics (see Section 4.2).

#### 4.1.2 Wildlife–Pollutant Attraction-like Interactions

While many chemical contaminants are expected to be repellents, some compounds can attract wildlife by interfering with sensory systems or by altering environmental cues used for habitat selection.^72^ This can result in “sink habitats” or even “ecological traps”, whereby organisms select suboptimal habitats where their exposure to harmful substances is heightened, and their fitness is consequently reduced. Some pesticides, for example, resemble insect pheromones, leading insects to mistake these chemicals for mating signals.^73^ Similarly, heavy metal pollutants can disrupt sensory system function, preventing organisms from detecting olfactory signals that might otherwise be avoided (e.g., predator cues).^74^ Furthermore, contaminated areas can be associated with modified local habitat characteristics (e.g., temperature, nutrient availability, sediment type), inadvertently making them more attractive to certain species. Wastewater effluents, for example, may attract fish due to nutrient-rich discharge and warmer temperatures, increasing their exposure to harmful contaminants.^75,76^

#### 4.1.3 Pollutant-Induced Behavioral Shifts

In addition to repulsion from or attraction to contaminated sites, chemical pollutants may also alter the spatial distribution of organisms and their subsequent exposure via effects on organismal behavior (i.e., without a spatially explicit response to the contaminant itself). Small- and large-scale movement patterns are sensitive to contaminants that affect neurological function, metabolism, and endocrine regulation, such as psychoactive pharmaceutical pollutants,^52,55,56,77^ endocrine-disrupting chemicals,^53,54,78,79^ and pesticides.^54,80,81^ As a small-scale example, chemicals can disrupt biological rhythms of exposed organisms, altering normal day–night activity cycles.^82,83^ As a larger-scale example, contaminants can alter travel distances, migration dynamics, and stopover durations.^84−86^ Contaminant-induced shifts in movement can, in turn, lead to altered subsequent exposures to the same or other pollutants (i.e., positive or negative feedback loops), by affecting the likelihood of encountering pollutants as well as the duration of exposure. Further, contaminant-induced effects on other behavioral traits may also influence the spatial distribution of organisms and their probability of future exposures. As an example, risk landscapes^87^ and social resistance (e.g., territoriality, within-group preferences)^88^ are known to be major barriers to movement in many species, and there is evidence that many chemical contaminants can modify behaviors that generate these barriers, such as territoriality, risk-taking, aggression, and social behaviors.^54,79,89−91^

#### 4.1.4 Individual-Specific Effects

Trait variation among individuals within a population may also determine the nature of individual exposure. For instance, several demographic characteristics (e.g., age, sex, body condition, reproductive status) are known to influence the spatial distribution of organisms in the environment (see Section 3). Similarly, individual differences in personality (e.g., foraging propensity, risk-taking behavior, sociality) and experience within populations can also mediate movement rates, space use, and habitat selection,^15,92,93^ suggesting that some individuals may be more likely to encounter contaminants than other individuals in the population.

Moreover, even when organisms are exposed to the same contaminant concentrations for the same duration, individual responses may still differ. Genetic and physiological differences can influence individual sensitivity to pollutants and their subsequent behavioral response. For instance, exposure to environmental levels of an antidepressant over two years homogenized movement behavior among individual male guppies (*Poecilia reticulata*), but no shift in the variation of female movement phenotypes was observed.^94^ Variation in metabolic rate, enzyme activity, and hormone regulation can also affect how contaminants are processed and detoxified, influencing the stress signals perceived by organisms and leading to the avoidance of, or attraction to, certain areas.^95^ Other traits have also been shown to influence the sensitivity of organisms to pollutants. Indeed, independent of body mass, social status influenced the bioaccumulation of the psychoactive pharmaceutical pollutant oxazepam and subsequent aggressive behavior in exposed brown trout (*Salmo trutta*).^96^ Taken together, this research highlights that where pollutants are spatially structured within an environment, individual differences in phenotypic traits (e.g., body condition, physiology, personality) likely mediate the nature and extent of exposure in the wild, and that this exposure can subsequently feedback to affect these same phenotypic traits. To our knowledge, the potential for individual phenotypic traits to influence exposure risk, moderate individual sensitivities, and feedback to influence those same phenotypes has not been empirically assessed.

### 4.2 Ecological and Evolutionary Consequences

Below, we illustrate several potential ecological and evolutionary consequences of spatiotemporal interactions between pollutants and organismal movement at the individual, metapopulation, and community levels. This overview is not intended to be exhaustive but instead highlights several key outcomes of spatiotemporal wildlife–pollution interactions that are seldom considered in ecotoxicology. It is also worth noting that many of the highlighted consequences likely have effects across multiple biological and spatial scales, which, for the sake of simplicity, we have not specifically illustrated here. While we have focused on movement, space use, and behavior, we acknowledge that many pollutants can exert a variety of ecological and evolutionary effects via other mechanisms (e.g., mutagenesis, direct mortality, disrupted organismal development, reproductive changes),^97,98^ which can also contribute to potentially adverse outcomes for wildlife populations.

#### 4.2.1 Individual-Level Outcomes

Likely consequences of pollution-induced changes in animal movement and space use are alterations in the rate and nature of conspecific encounters (i.e., intraspecific interactions). For example, pollutants that act as repellents or attractants may decrease or increase intraspecific encounter rates via changes in local population density. Likewise, pollutants that increase movement rates may similarly heighten the likelihood of encountering conspecifics (and *vice versa*). Changes in encounter rates and local population densities could lead to shifts in the strength/direction of both natural and sexual selection within the population via changes in resource (e.g., food and shelter) competition, disease, and social information transmission, as well as altered mating dynamics (e.g., inter- and intrasexual competition). For example, in brown trout, methamphetamine (a common psychoactive pollutant) has been reported to cause a spatial attraction of individuals to methamphetamine-polluted zones,^99^ while also reducing individual movement^99,100^ and increasing conspecific aggression,^101^ in combination creating conditions that would likely disrupt the local ecological interactions of brown trout populations. In addition, pollution-induced changes in wildlife movement and space use could alter interspecific interactions, including changes in predation,^102^ pollination,^103^ and parasitism. For example, mummichog killifish (*Fundulus heteroclitus*) from metal-contaminated environments exhibit slower movement rates, resulting in a decreased ability to capture prey and an increased susceptibility to predation themselves.^102^

These interactions may be further complicated, where individuals differ in their response to the pollutant, thus altering the distribution of movement phenotypes within the population. Where such traits are associated with fitness (e.g., via predation susceptibility), this will reduce the variation available for selection to act upon within the population. However, variation in pollutant sensitivity is not necessarily fixed; selection on toxicity-mediating genes can result in populations evolving tolerance (or resistance) to chemical pollutants, as seen in killifish (*Fundulus* sp.)^104,105^ and numerous other species that have evolved tolerance to pesticides.^98^ It may seem like an overwhelming challenge for ecotoxicology to incorporate these complex interactions between individual physiological sensitivity, pollution-induced changes in movement traits, organismal fitness, and adaptive tolerance in spatially and temporally dynamic environments, but in many ways, it is necessary if we are to accurately predict and assess the impacts of pollution on wildlife.

#### 4.2.2 (Meta)population-Level Outcomes

Pollutant-induced changes in movement and space use also have clear consequences for the ecoevolutionary dynamics of (meta)populations. While avoiding exposure can be individually a more advantageous strategy than enduring the costs of chemical toxicity and depuration,^106^ avoidance behavior also acts as a barrier to movement, resulting in habitat fragmentation, potentially affecting gene flow and population connectivity.^58,64^ Even in the absence of direct avoidance, where pollutants alter dispersal-related traits—as seen in freshwater isopods (*Asellus aquaticus*) following sublethal insecticide exposure^107^ —there are likely changes in population growth rates via emigration and immigration and subsequent gene flow. For several bat star (*Patiria miniate*) populations, pollution from stormwater runoff and wastewater effluent has been shown to act as a barrier to dispersal and gene flow, leading to reduced genetic diversity at highly contaminated sites.^108^

Differential sensitivity to pollutants may also influence gene flow between populations via specific changes in allele frequencies rather than changes in the absolute number of migrants. Research in alpine whitefish (*Coregonus* sp.) and marine invertebrates (*Peramphithoe parmerong*) has demonstrated genetic variation in tolerance to pollution for endocrine-disrupting pollutants^109^ and copper pollution,^110^ respectively. In cases where tolerance and avoidance of pollutants are genotype-dependent, this may lead to pollutant-induced spatial sorting of genotypes (and phenotypes). For instance, chemical pollutants were found to serve as genotype-dependent dispersal barriers in Mediterranean mussels (*Mytilus galloprovincialis*), leading to substantial population genetic differences over short distances.^111^ Conversely, shifts in space use due to preferences (either direct or indirect) for highly contaminated sites (e.g., refs (75 and 76)) or avoidance of polluted areas (e.g., ref (8)) could also increase interbreeding and hybridization between previously isolated groups, resulting in greater genetic diversity within populations.

#### 4.2.3 Community-Level Outcomes

Pollutant-induced changes in movement and space use at the individual level can scale up to impact community and ecosystem dynamics. For example, shifts in predator–prey interactions caused by chemical pollutants (e.g.,refs (55 and 102)) have been shown to restructure food webs.^112^ Contaminants can also transfer through trophic interactions and even biomagnify, leading to complex exposure patterns for species across ecosystems.^113,114^ Furthermore, species often exhibit varying sensitivities to chemical pollutants (e.g., ref (115)), and in some cases, community composition may moderate responses to contaminant exposure.^115,116^ For instance, zebrafish (*Danio rerio*) and freshwater shrimp (*Atyaephyra desmarestii*) demonstrated different spatial avoidance behaviors when tested independently versus together in response to copper pollution.^116^

## 5 Ways Forward

Predicting the outcome of dynamic interactions between pollutants and organisms across different scales of biological complexity is inherently challenging and requires detailed knowledge of both organism- and environment-specific factors. Nevertheless, it is imperative to advance research on spatiotemporal exposure risks to accurately predict the ecological and evolutionary impacts of chemical pollution. While ecotoxicology has a relatively long history of conducting laboratory-based contaminant attraction/avoidance studies,^117−119^ spatial and temporal variation are still not widely incorporated, and the scope of these studies has often been limited. For instance, few studies have investigated whether individual variation within populations in behavioral and movement traits predicts an organism’s level of attraction to, or avoidance of, contamination.

**Figure 2:**
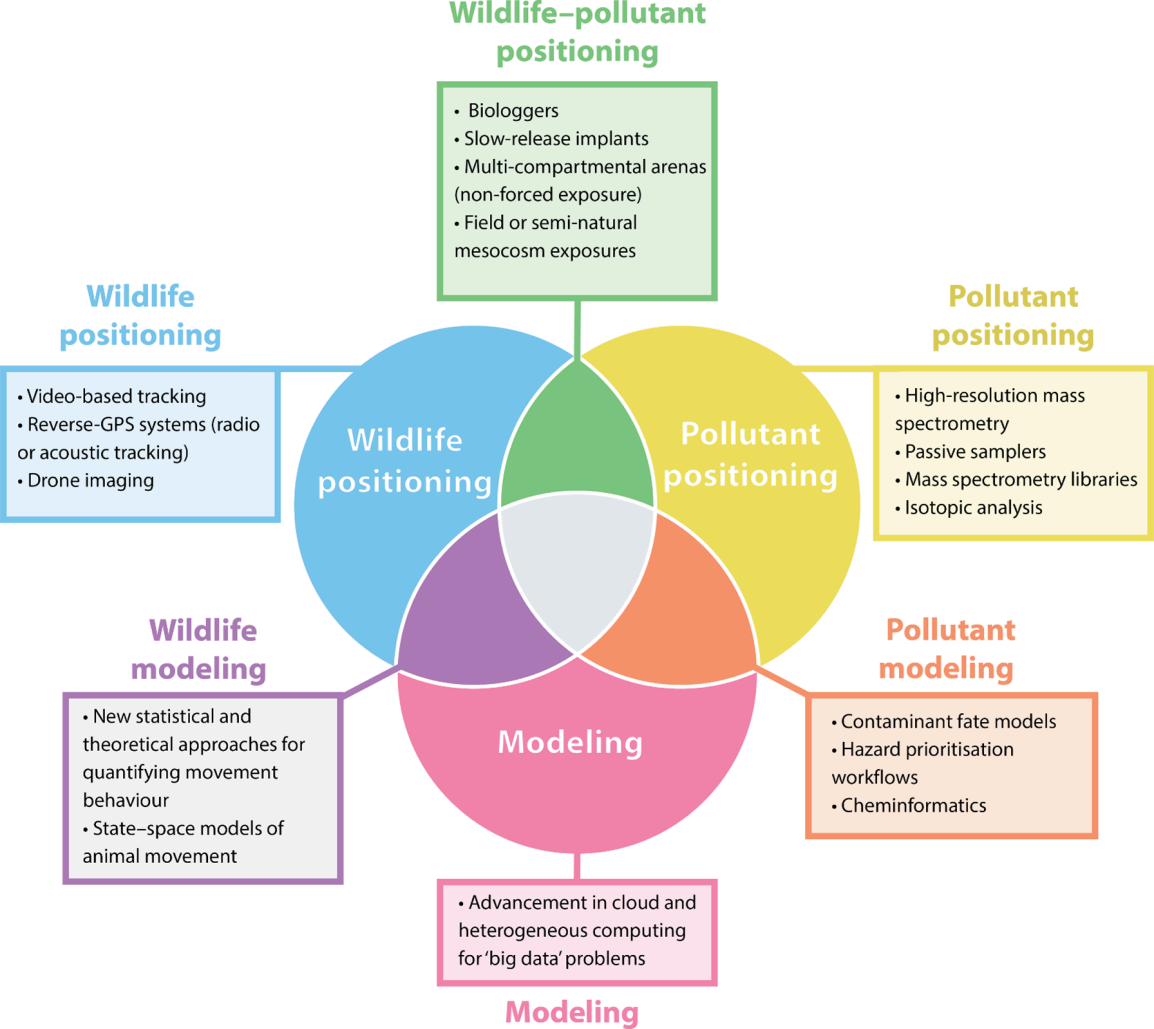
Recently developed and established methodological and technological approaches that can facilitate the study of the spatiotemporal dynamics of wildlife–pollution interactions: wildlife–pollutant positioning;^68,120−122^ pollutant positioning;^123−126^ pollutant modeling;^127−129^ modeling;^130^ wildlife modeling;^131,132^ wildlife positioning.^133−135^ Approaches that can combine all of these different techniques (e.g., gray center of the Venn diagram)—such as agent-based models that incorporate empirical data from the spatiotemporal distribution of both wildlife and pollutants—may be particularly promising in predicting the outcomes of spatiotemporal dynamic wildlife–pollutant interactions.

To advance this field, it is necessary to incorporate the spatiotemporal variability of pollutants and the movement patterns of wildlife into existing research frameworks as well as increasing crosstalk between related disciplines. In this regard, recent methodological and technological advancements in ecotoxicology, analytical chemistry, and animal tracking, as well as artificial intelligence and computational modeling, provide unprecedented opportunities to address these complexities (Figure 2). Using these recent advancements, we outline a three-pronged approach to guide future research in this area: *in silico* modeling, laboratory experiments, and semifield and field studies. It is important to highlight that such approaches may not be equally applicable to all environmental matrices. For example, *in silico* modeling and field-based experiments may be much more feasible in small freshwater lentic ecosystems (e.g., lakes) when compared to large marine systems (e.g., seas and oceans). Nevertheless, we believe that such approaches may provide insights into the nature of spatiotemporal interactions between organisms and pollutants across a range of habitat types.

### 5.1 *In Silico* Tools

While verbal and conceptual models are a key first step in describing dynamic interactions between contaminants and organisms (Figure 1), computational approaches are required to predict the outcomes of such interactions over time.

Agent-based modeling (ABM) is a key tool to investigate how wildlife will respond to changing environmental conditions—including contaminants—given that these models are able to incorporate the adaptive movement ecology of animals inhabiting a changing landscape.^136^ As an example, ABM approaches incorporating individual movement and life-history traits in combination with pesticide application schedules have been used to predict spatial patterns of pesticide exposure, as well as subsequent population dynamics.^137^ Despite their utility, ABMs have rarely been applied to understand complex interactions and feedback between spatiotemporally dynamic contaminants and animal movement, particularly in terms of within-population variation in movement. Such approaches are increasingly feasible given the increase in modern computing power and the development and refinement of contaminant fate models.^129^ Integrating spatial and temporal information on contaminant concentrations at a local scale into ABM approaches will be critical in predicting how individual variability in movement and behavior affects exposure to contaminants, providing insights into the potential long-term effects on population dynamics.

However, these ABMs need to be parametrized and validated based on empirical data, emphasizing a need for more research into the spatiotemporal variation of contaminants in natural systems. To this end, *in silico* tools, such as advanced data-driven computational models, supervised machine learning algorithms and artificial intelligence tools, molecular networking, and chromatographic retention time prediction, have been developed to help identify and predict the effects of thousands of potential contaminants that are detected in environmental and biological matrices using high-resolution mass spectrometry (HRMS).^138−141^ With such approaches, concentration,^142^ toxicity,^143^ and endocrine-disrupting activity^144^ can be derived from the chemical structure.^138,145^ Feature-based molecular networking (FBMN) is a high-throughput tool that can identify related chemicals in a sample, indicating potential transformation or degradation pathways of labile substances.^146^ These *in silico* analytical chemistry tools, coupled with high sensitivity profiling methods, will be essential if we wish to determine the spatial and temporal scale of pollution at a high resolution.

### 5.2 Laboratory Experiments

Conventional studies in ecotoxicology typically expose organisms to contaminants within spatially restricted compartments (e.g., containers, aquaria) and/or under temporally consistent exposure conditions (acute exposure, 24–96 h; chronic exposure, several days to months^12,13^). While useful for testing the toxicity and concentration thresholds of different chemicals, this approach limits the organisms’ ability to exhibit their full range of behaviors, such as the capacity to move away from contaminated areas. Many laboratory studies have demonstrated that animals actively avoid contaminated habitats when given the option.^147−149^

To overcome these limitations, multicompartmental arenas^69,117^ and steep gradient assays^150^ offer effective alternative designs. These designs incorporate ecological complexity into laboratory experiments while allowing for more spatial and temporal heterogeneity in exposure conditions.^151^ By combining these experimental designs with consumer-grade video cameras and freely available animal tracking software, researchers can obtain high-resolution (spatial and temporal) measurements of individual and group behaviors—see Bertram et al.^12^ for a list of tracking software options. This approach also allows for the quantification of individual variation in movement and within-population variation in exposure risks under different ecological and chemical contaminant scenarios, which are ideally informed by spatially explicit field sampling (Section 5.3).

To further refine these experiments, the integration of environmental variables that mimic real-world conditions is crucial. For example, creating gradient-based exposure scenarios that simulate the gradual increase or decrease of contaminant concentrations across a landscape can reveal how animals detect and respond to changing contamination levels.^64^ Similarly, incorporating dynamic elements such as fluctuating contaminant levels or introducing other ecological pressures (e.g., predation risk) can offer insights into how animals balance their responses to multiple stressors, providing a more realistic prediction of their responses in natural environments.^69,152^ Further, incorporating mixture exposures based on observed environmental (co)occurrences and predicted biological interactions (e.g., via slow-release chemical mixture implants^153^ or exposure to real-world wastewater effluents^154^) would more accurately reflect environmental conditions and could elucidate the potential interactive effects of different contaminants.

In all cases, it remains essential for future research in this area to adhere to fundamental principles of ecotoxicology wherever possible.^12^ This includes aspects of sound experimental design and quality control such as adequate replication and standardization, the use of appropriate controls and study designs, accurate measures of exposure concentrations and relevant environmental parameters, and the use of appropriate statistical techniques. Incorporating such principles will be key in enhancing research credibility and reproducibility, which is particularly relevant for research that aims to inform chemical risk assessments and regulation.^12^

### 5.3 Field Studies

Laboratory studies are invaluable for understanding the underlying mechanisms of contaminant effects and rapidly generating predictions that can be applied to real-world scenarios. However, the outcomes of laboratory experiments often diverge from field observations due to the inherent limitations of replicating the complexity of natural systems within controlled environments.^155−157^ Thus, spatially explicit water sampling and field studies are necessary for characterizing complex exposure scenarios and monitoring the spatial and temporal overlaps of chemical contaminants and animal populations.

Advancements in mass spectrometry libraries and computational tools, combined with spatially explicit sampling regimes, are improving the identification and characterization of contaminant distributions in the field.^127^ These tools allow researchers to capture the fine-scale spatial and temporal variability of contamination in nature, offering a more precise and comprehensive understanding of the true exposure risks to wildlife populations. With that being said, the process of field-validated ecotoxicological experiments is costly in terms of financial commitment and personnel time. Therefore, the careful selection and prioritization of chemicals that are predicted to have environmental implications are key to reducing these costs. As mentioned above, *in silico* modeling can be used to help select chemicals with predicted toxicity and to highlight transformation products that may also contribute to the overall risk to environmental health. To elucidate potentially harmful substances from complex environmental matrices, effects-directed analysis is a powerful technique that has benefited by improved HRMS techniques to simultaneously identify chemicals and perform *in vitro* toxicity tests.^158^

Moreover, advances in remote-sensing technologies, such as acoustic telemetry and global positioning systems (GPS), have revolutionized our ability to quantify the behavior and movement of animals in their natural habitats.^17^ These tools, when combined with spatially explicit field sampling, enable researchers to map the spatial distribution of animal populations, track their movements, and assess their potential exposure to contaminants. Targeted exposure devices, such as slow-release implants, are another emerging tool that can be used to study exposure under field-realistic settings.^86,121,153^ Targeted exposure devices can be used to isolate chemical exposure to specific individuals in the field, while holding spatial exposure elements constant (i.e., the animal remains homogeneously exposed while still moving freely) to disentangle complex wildlife–pollutant spatial interactions.^121^ In combination, such an approach offers unprecedented opportunities to understand the impacts of contaminants on (meta)populations and community-level processes by delivering near-continuous data on individual movements and ecological interactions (e.g., social dynamics, predator–prey relationships).^159−161^

Here, we categorize pollutant–animal spatial interactions and conceptualize a simple dynamic feedback model that may result from such interactions. We identify potential ecological and evolutionary consequences and highlight key areas of uncertainty. We recognize that incorporating these spatial interactions in experimental and observational work generates logistical challenges but emphasize that it is becoming ever more achievable, with advances in *in silico* modeling and prediction techniques, artificial intelligence, and laboratory- and field-based animal-tracking technologies, as well as the rapid advances in high-throughput and sensitive analytical chemistry approaches. We contend that considering and incorporating wildlife–pollutant spatiotemporal interactions in ecotoxicology will improve our ability to assess and predict the risk of contaminants to wildlife.

## Data Availability

There are no new data associated with this article.
